# Evaluation of the Long-Term Success and Patient-Related Outcomes of Zygomatic Implants in Atrophic Maxillary Ridges

**DOI:** 10.7759/cureus.64280

**Published:** 2024-07-10

**Authors:** Rahul Koppaka, Nabeel Ahmed, Urvi R Echhpal

**Affiliations:** 1 Prosthodontics and Implantology, Saveetha Dental College and Hospitals, Saveetha Institute of Medical and Technical Sciences, Saveetha University, Chennai, IND

**Keywords:** full mouth rehabilitation, full mouth implants, titanium implants, maxilla atrophy, zygoma implants

## Abstract

Introduction

Zygomatic implants (ZIs) have emerged as a promising option for rehabilitating completely edentulous patients with severe maxillary atrophy. These implants anchor into the zygomatic bone, bypassing the need for extensive grafting procedures. Success rates in dental and craniofacial implant surgeries can be influenced by several surgical factors, including suture techniques, flap design, and treatment planning. The research aimed to present the clinical outcomes and complications in individuals with severely resorbed maxillae who underwent prosthodontic rehabilitation using the Quad Zygoma Protocol (QZP) and the Anatomy-Guided Approach (AGA), focusing on long-term assessment.

Material and methods

Data for this retrospective study were extracted from the institution's patient database, involving a meticulous review of patient records. This comprehensive examination encompassed demographic data, preoperative assessments, details of surgical procedures, postoperative complications, and subsequent follow-up evaluations. Patients with severe maxillary bone deficiencies resulting in complete edentulism, due to inadequate bone quality and quantity in both anterior and posterior regions, were selected for inclusion. Exclusion criteria were applied to individuals with incomplete records or insufficient follow-up data, as well as those who underwent alternative treatment modalities or presented with comorbidities potentially impacting implant outcomes. The selected patients underwent treatment utilizing the QZP, with each participant subjected to a minimum three-year follow-up period. The implant survival rate, prosthetic success, complications, and Oral Health-Related Quality of Life using the OHIP-14 questionnaire were assessed.

Results

At the end of the follow-up period involving 12 patients (eight men, four women) with 43 ZIs - 37 from Neodent, four from Nobel Biocare, and two from Norris - with a mean duration of 4.3 years (range: 1.2-5.4), the overall success rate stood at 99.08%, with only 1 out of 42 implants failing. All patients received immediate loading with an acrylic prosthesis, proving effective in 98.2% of cases. The most common issues observed were localized soft tissue inflammation (35.7%) and sinus inflammation (12.5%), occurring after mean follow-up periods of 1.2 and 3.5 years, respectively. In 12 patients, the mean score of the OHIP-14 questionnaire was 1.6 ± 2.6, with a follow-up period of 5 ± 0.6 years.

Conclusion

The QZP has consistently demonstrated excellent long-term success in restoring severely reduced maxillary structures. An immediate loading approach could aid in stabilizing ZIs through cross-arch support.

## Introduction

The maxilla, an essential component of the facial structure, plays a pivotal role in aesthetics, speech, and mastication. However, factors such as tooth loss, trauma, congenital defects, or tumor resections can lead to severe bone resorption over time [1]. Restoring severely diminished upper presents a significant challenge in implant dentistry, as conventional implant placement becomes difficult due to insufficient bone height and width. Patients dealing with this predicament often face prolonged treatment times, significant morbidity related to donor sites, and additional costs associated with biomaterials. Furthermore, prosthodontic rehabilitation may be delayed, necessitating two-stage procedures when primary implant stability is not achieved or when hard tissue reconstruction is required before implant placement [2]. Traditional implant techniques often need to be revised when dealing with inadequate bone volumes. 

Zygomatic implants (ZIs) have emerged as a promising option for rehabilitating completely edentulous patients with severe maxillary atrophy. These specialized implants anchor into the zygomatic bone, bypassing the need for extensive grafting procedures. The concept of using the zygoma as an anchorage site was initially explored by Prof. PI Brånemark in 1988, utilizing custom-made, extended-length conventional implants [3]. Aparicio et al.’s [4] literature report further highlighted this technique, demonstrating the stabilization of a graft in the pre-maxilla using the zygomatic process of the maxilla for anchorage. ZIs provide stability and support for fixed prostheses, allowing patients to regain function, aesthetics, and comfort. They are particularly indicated for patients with severe maxillary atrophy, lack of posterior maxillary bone support due to significant sinus pneumatization, and severely resorbed maxillary posterior alveolar ridges. The International Team for Implantology (ITI) consensus report on ZIs outlines the criteria for the quad zygoma approach, which involves using two ZIs bilaterally in a balanced front-to-back configuration as an alternative when traditional implants are not suitable for both the front and back of the upper jaw. 

The Quad Zygoma Protocol (QZP), initially introduced by Duarte et al. [5] in 2007, builds upon the Brånemark intrasinus technique. Subsequent elaboration by Stiévenart and Malevez [6] in 2010 further refined this approach. Additionally, Davó et al. [7] explored the Anatomy-Guided Approach (AGA), achieving notably high success rates. A fairly new systematic review, including 24 studies, compared the original surgical technique (OST) pioneered by Brånemark against the AGA for rehabilitating severely reduced upper jaws [8]. The findings indicated that OST had higher implant survival rates (ranging from 90.3% to 100%) but also higher rates of complications, including sinus inflammation (9.3% vs. 4.4%), soft tissue contamination (7.5% vs. 4.3%), paresthesia (10.8% vs. 4.3%), and oroantral fistulas (4.6% vs. 0.6%). Conversely, data from a randomized controlled trial showed that immediately prosthetically loaded ZIs had fewer prosthetic and implant failures and achieved functional loading more quickly compared to conventional implants inserted in augmented bone after an extended period. Additionally, patients undergoing ZI rehabilitation experienced improved prosthetic restoration in terms of both function and anatomical appearance, thanks to a less invasive surgical approach [9]. The precise positioning of ZIs within or extrinsic to the sinus is influenced by specific anatomical features of the maxillary-zygomatic complex, aiming for proper emergence at the crest and alignment with prosthetic objectives [10]. Success rates in dental and craniofacial implant surgeries can be influenced by several surgical factors, including suture techniques, flap design, and treatment planning [11-14].

However, despite their success, there are still important considerations and potential complications associated with zygoma implants. In this context, understanding the role of ZIs and addressing the challenges they present becomes crucial for successful rehabilitation.

The primary aim of this retrospective study was to evaluate the clinical outcomes, complications, and influence on Oral Health-Related Quality of Life (OHRQoL) associated with the QZP over a five-year follow-up period. By analyzing a comprehensive dataset, this study aimed to contribute to the current understanding of the therapeutic effectiveness of quad ZIs in patients with severely compromised upper jawbones. The null hypothesis states that zygoma implants are not a favorable treatment option for patients with atrophic maxilla.

## Materials and methods

Study design

This retrospective study utilized patient records from a dental institution, Saveetha Dental College, Chennai, India, specializing in implant dentistry. The study protocol was approved by the Scientific Review Board (SRB/SDC/PROSTHO-2103/23/143).

Preoperative evaluation

A panoramic X-ray, along with a cone beam computed tomography (CBCT) or CT scan, was conducted to assess the extent of atrophy in the upper jaw. Prosthetic planning, driven by the desired outcome, was carried out using specialized software (3Shape Implant Studio; 3Shape, Copenhagen, Denmark). The degree of maxillary atrophy was classified using the Cawood and Howell classification system. A physical examination was performed to evaluate mouth opening, occlusal relationships, intermaxillary distance, and the condition of the lower jaw teeth. Patients with periodontal issues in the lower jaw, where impacted teeth were planned to be retained, received antibiotic treatment before surgery [15]. Cases of sinusitis were addressed by an otorhinolaryngologist. Diagnosing sinusitis relied on clinical symptoms like facial pain/pressure, congestion, nasal obstruction, reduced sense of smell, nasal discharge, dental pain in the upper jaw, fever, bad breath, ear pain, and further assessments as deemed necessary by the specialist, including anterior rhinoscopy, sinuses transillumination, and relevant microbiological or imaging studies. Before initiating implant therapy, complete resolution of all symptoms was required [16].

Surgical protocols and provisionalization

**Figure 1:**
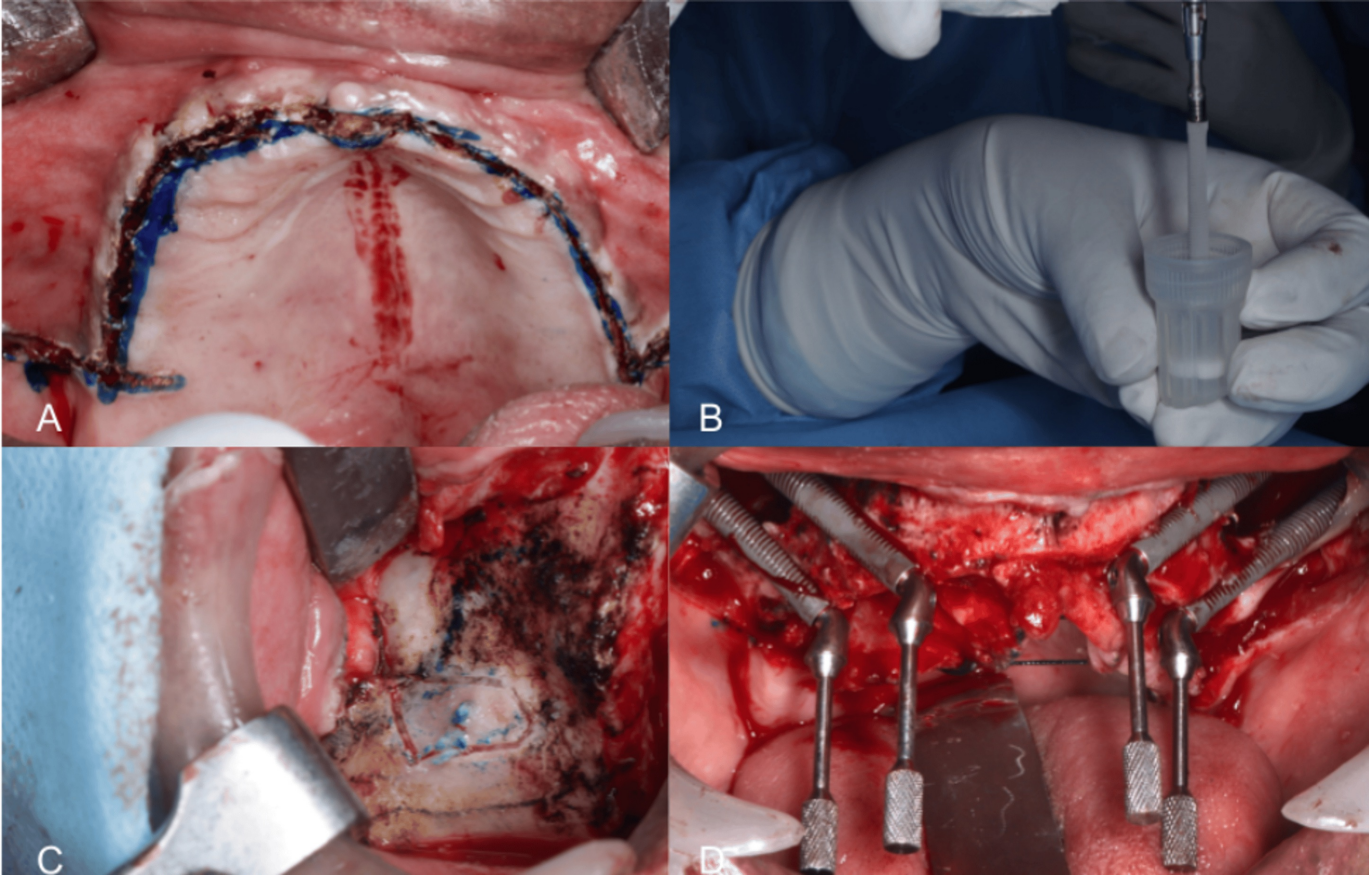
A) Incision; B) Zygoma implant; C) Sinus lift approach; D) Multi-unit abutments in position

A midline crestal or palatal incision (Figure 1A) was employed to uncover the zygomatic area, followed by vertical releasing cuts towards the back of the infra-zygomatic bone and towards the front of the surgical area [17]. The entry points for the intraoral sections of the posterior and anterior implants should align with or be close to the upper edge of the remaining jawbone crest, corresponding to the canine/lateral incisor region for the front implant and the first molar/second premolar area for the rear implant. Neodent Zygoma GM, NobelZygoma series 45 (Nobel Biocare, Gothenburg, Sweden), and Norris Zygoma implants (Norris Medical, Kiryat Ono, Israel) were the ZI systems employed (Figure 1B). To determine the right length of the implant, a succession of drills and a depth indicator were utilized to enter the dentoalveolar process and the zygoma bone [18]. The sequence of drilling only used three burs: one 2.9 mm round bur and cylindrical bur with diameters of 2.9 mm (Nobel Biocare, Straumann, and Norris) and the last 3.5 mm. To prevent overheating, drilling was conducted with constant water irrigation. Preserving the integrity of the sinus membrane for the intrasinus path involved a careful dissection of the sinus membrane through a small lateral bone window (Figure 1C). Critical anatomical structures like the eye cavity or infra-temporal fossa were avoided by positioning the ZIs at the level of the zygomatic bone. While implants were typically placed using a handpiece, final adjustments for optimal placement were manually carried out. After implant placement, a multi-unit abutment was utilized, offering straight and angulated connections (Figure 1D).

**Figure 2:**
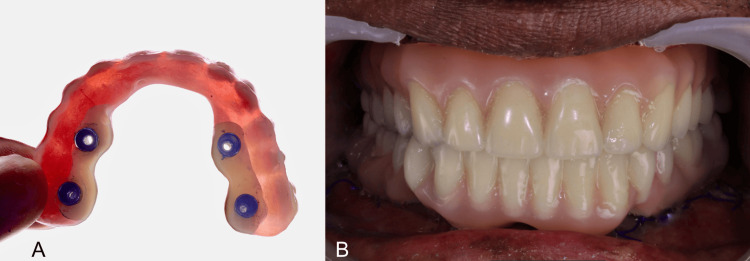
Temporization with existing complete denture: A) Intaglio surface; B) Temporary prosthesis in occlusion

After suturing, the pre-fabricated prosthesis was used to pick up the temporary cylindrical abutments to design the immediately loaded prosthesis. This involved using non-engaging temporary cylindrical abutments placed in the respective positions of the prosthetic component, and pick-up was done using pattern resin (PATTERN RESIN™ LS). Temporary prostheses were crafted by modifying the pre-fabricated prostheses and were inserted within 24 to 48 hours post-operatively (Figure 2) [19].

Patients underwent check-up appointments at one week, one month, three months, and six months after loading the temporary prosthesis. At the six-month mark, a new impression was taken to craft the final fixed prosthesis supported by implants. In a few cases, adjustments were made to the acrylic bridge to transform it into a permanent prosthetic solution [20]. Subsequently, patients were monitored annually after receiving their final prosthesis. Additionally, specific radiographic studies were conducted based on the attending doctor's judgment.

**Figure 3:**
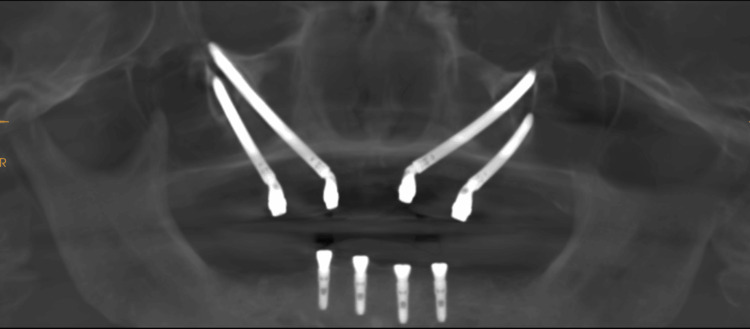
Post-operative CBCT panorama CBCT: Cone beam computed tomography

During every follow-up appointment (at one month, three months, and then every six months), panoramic radiographs were routinely taken when patients displayed symptoms suggestive of issues like orofacial discomfort, swelling, local inflammation, signs of infection, or sinus-related problems. CT or CBCT scans (Figure 3) were used as needed. The prosthesis was removed either annually or whenever a patient encountered a problem necessitating its removal.

Outcomes assessed

Patient records were reviewed to collect the following data: demographic information (age, sex), postoperative complications (such as infection, implant failure, and soft tissue complications), long-term implant survival rates (5 ± 0.6 years), and OHRQoL assessments.

Statistical analysis

The gathered data were statistically analyzed utilizing IBM SPSS Statistics for Windows, Version 20 (Released 2011; IBM Corp., Armonk, NY, USA) to ascertain the prevalence of isolated cleft lip across different age brackets, genders, and primary cleft lip operations. Additionally, the study aimed to determine if there existed any statistically significant correlation between gender, age, and the technique employed. The analysis used IBM SPSS Version 20 to explore these factors, employing Pearson's Chi-square test. Subsequently, the outcomes were compiled and illustrated using bar charts for visual representation.

## Results

The study employed a non-probability convenience sampling method. The records consisted of individuals with severe atrophy in their upper jaw seeking fixed prosthetic solutions. Information regarding the quad ZI surgical approach was also gathered. Data spanning from May 2018 to December 2023 were collected and organized. To prevent sample bias, all available data were included without any sorting process. Subsequently, the analysis was performed, and any suppressed or incomplete data were excluded. An external reviewer then validated the data. The study encompassed a total of 12 individuals, with males comprising 66.6% and females 34.4%, aged between 21 and 56 years old (Table 1).

**Table 1:** Descriptive data of the participants

Gender	Patients n (%)	Implants n (%)	Mean age at enrolment (years ± SD)	Hypertension (yes:no)	Diabetes (yes:no)	Smoking (yes:no)	Follow-up (months ± SD)	Failures n (%)
Male	8 (66.6)	32 (68.08)	60.2 ± 9.26	7:1	6:2	6:2	27.2 ± 6.48	0 (0)
Female	4 (33.3)	15 (31.9)	58.4 ± 8.41	0:4	2:2	0:4	25.05 ± 7.62	0 (0)
Total	12 (100)	47 (100)	59.1 ± 8.68	9:3	8:4	6:6	36.6 ± 5.34	0 (0)

The main focus was on the survival of ZIs, with secondary attention given to identifying any post-operative complexities. Postoperative sinus inflammation, determined by clinical symptoms like breathing difficulties, swelling, redness, or tenderness, was treated with the help of antibiotics, NSAIDs, nasal decongestants, and menthol inhalations. Surgical intervention such as functional endoscopic nasal surgery (FENS) or implant removal wasn't carried out for patients with sinusitis. The study also examined the stability of the implant and soft tissue health, gauged using bleeding on probing and a modified gingival index scoring system, where different scores indicate varying levels of bleeding. The OHRQoL was assessed using the self-administered OHIP-14 questionnaire at least six months following the loading of the definitive prosthesis. An independent assessor, not previously involved in the study, conducted all the measurements. The OHIP-14 is a 14-item tool that examines the individual's perception of the social impact of oral health issues and overall well-being. Each item is rated on a scale from 0 to 4 (0 = never, 1 = hardly ever, 2 = occasionally, 3 = very often, 4 = fairly often), where 0 indicates no impairment and 4 indicates the highest level of impairment. The total score, ranging from 0 to 56, is the sum of all individual item scores.

Age and gender

**Figure 4:**
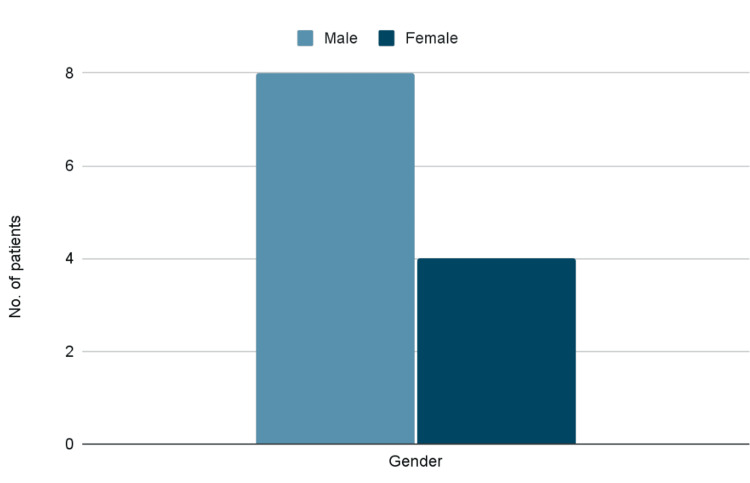
Bar graph depicting the gender variation Eight male patients and four female patients were included in the study

**Figure 5:**
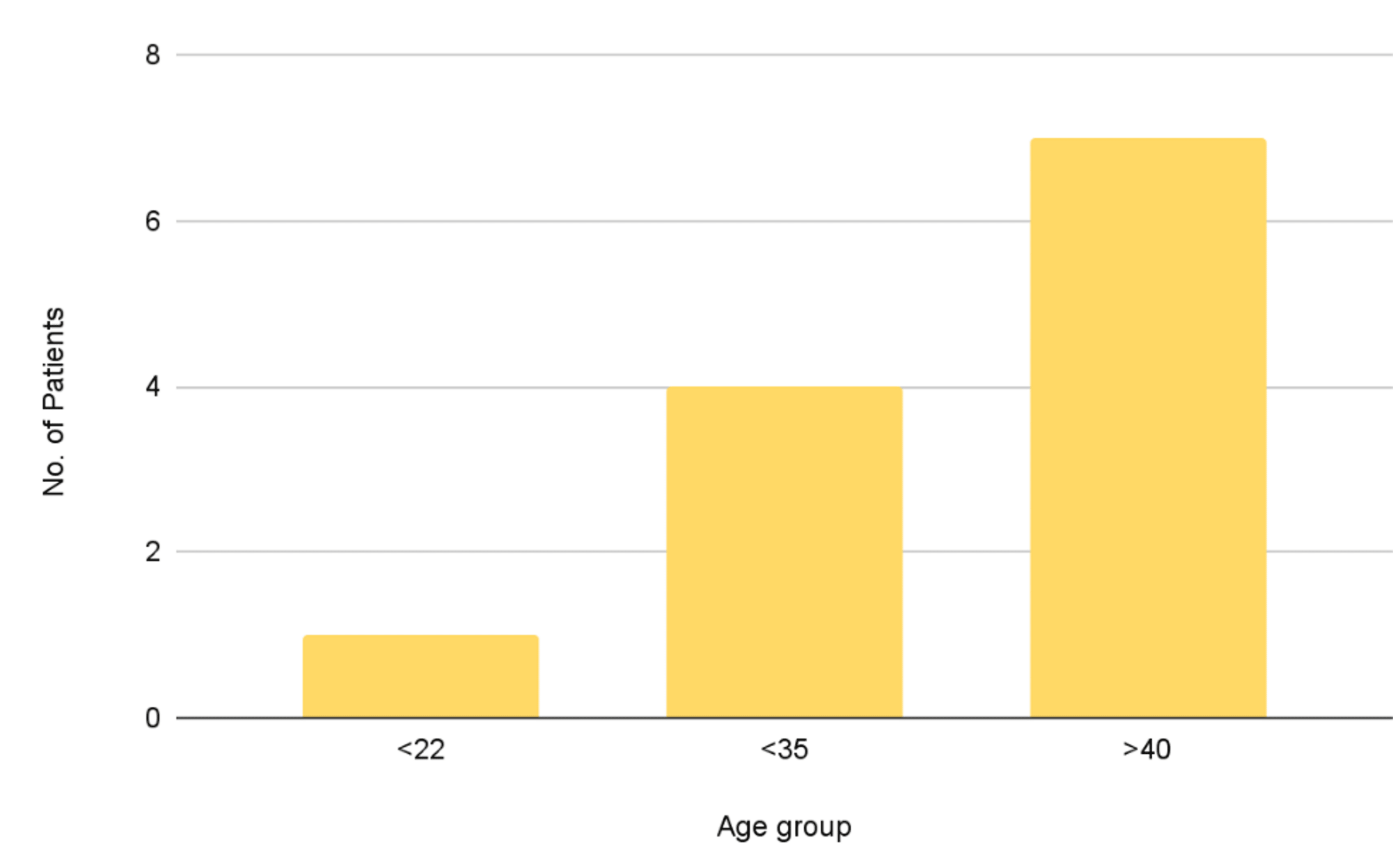
Bar graph depicting the age group frequency One patient was under 22 years of age, four were below 35 years and the remaining seven were above 40 years of age

In the group of 12 patients, there were four (34.4%) females and eight (66.6%) men (Figure 4). Among these patients, one individual was under 22 years old, four patients were below 35 years, and seven patients were above 40 years of age (Figure 5).

Patient and implant baseline characteristics

The median age of patients during surgery was 46, with a deviation of 9.4 years (ranging from 21 to 64). Among the patients, nine individuals (75%) underwent the placement of four ZIs using the quad zygoma technique, while two patients (17%) received two implants, and one patient (8.3%) received three ZIs. The majority of implants (35 in total) were positioned along the maxilla wall, with their implant head placement either on the ridge (four implants), vestibular (three implants), or palatal (28 implants). Additionally, 12 implants were inserted with an extra sinus, with their implant head placement either vestibular (four implants) or on the ridge (eight implants). The final recorded insertion torque was available for all 47 implants, averaging at 35 Ncm with a deviation of 6.8 Ncm (ranging from 35 to 55) (Table 2).

**Table 2:** Baseline characteristics ASA: American Society of Anesthesiologists

Characteristics		n (%)	n (evaluated)
Patients			12
Gender	Female	4 (33.3)	12
	Male	8 (66.66)	
Oral hygiene status	Excellent	0 (0)	12
	Good	5 (41.6)	
	Acceptable	4 (33.3)	
	Poor	3 (25)	
Smoking status	Yes	2 (16.6)	12
	No	10 (83.3)	
Patient physical status	ASA I	8 (66.6)	12
	ASA II	4 (33.3)	
Health history	Diabetes	0 (0)	82
	Cancer	0 (0)	
	Rheumatoid arthritis	2 (2.4)	
History of periodontitis	No	6 (50)	12
	Yes, treated	4 (33.3)	
	Yes, not treated	2 (16.66)	
History of sinusitis	No	9 (75)	12
	Yes, treated	3 (25)	
	Yes, not treated	0 (0)	
Indication	Partially edentulous	3 (25.0)	12
	Fully edentulous	9 (75)	
Implants/implant sites			47
Implant length (in mm)	42.5	17 (36.17)	47
	45	8 (17.6)	
	47.5	12 (25.5)	
	52.5	10 (21.2)	
Bone quality	Soft	1 (10.4)	12
	Medium	8 (70.9)	
	Hard	3 (18.7)	
Bone grafting before implant surgery	Yes	0 (0)	12
	No	4 (40)	
Bone grafting at implant surgery	Yes	0 (0)	47
	No	0 (0)	
Loading protocol	Immediate	11 (91.66)	12
	Early	9 (5.1)	
	Delayed	2 (1.1)	

Clinical follow-up and outcome measures

Among the 47 implants studied, all were deemed stable during the final follow-up, resulting in a 100% survival rate. Post-operative complications were examined in four implants among five patients, revealing sinusitis in four implants across three patients, mucosal complications around three implants in one patient, and prosthetic complications in two patients; no fistulas were reported. Specifics regarding sites with complications are outlined in Table 2. Soft tissue assessment occurred at a single center, involving three implants within one patient. The evaluations indicated excellent tissue health: 41 implant sites (85.5%) showed no bleeding upon probing and had a gingival index score of 0, while the remaining six sites (15.5%) exhibited a gingival index score of 1 (indicating sporadic bleeding spots) across five patients (Table 3).

**Table 3:** Complications, treatment, and outcome

Complications	n (%)	Treatment	Outcome
Zygoma implants (n = 43)			
Loss of osseointegraation	0 (0)	Nil	Success
Orosinusal fistula	4 (9.3)	Soft tissue graft	Success
Facial fistula	0 (0)	Nil	Success
Mucosal recession	3 (6.9)	Soft tissue graft/implantoplasty	Success
Patients (n = 12)			
Sinusitis	3 (25)	Pharmaceutical treatment	Success
Prosthetic	2 (16)	Repair/replacement	Success

OHRQoL

Of the 12 patients, 11 (91.6%) completed the OHIP-14 questionnaire. The average score was 1.6 ± 2.6, with a mean follow-up period of 5.0 ± 0.6 years (range: 4.5-5.5 years) after surgery. Patients with a follow-up of more than three years (n = 4) had lower mean scores compared to those with a shorter follow-up period (n = 7) (1.9 ± 1.7), though the differences were not statistically significant (p = 0.24).

## Discussion

Suture techniques, flap design, and treatment planning are among the surgical factors that can affect the success of dental and craniofacial implant surgeries [21-23]. This retrospective study showcases the impact of QZP in restoring edentulous individuals with significantly resorbed upper jaws. It highlights a notable survival rate for ZIs loaded promptly, delivering satisfactory prosthetic outcomes. The most commonly seen issues observed were local orofacial inflammation and sinusitis, both effectively resolved without compromising the overall results. These favorable long-term findings contribute to substantiating the advantages of QZP compared to removable dentures or traditional implant-retained restorations for atrophic maxillae, which at times necessitate complicated and phased bone grafting procedures. Immediate loading of the implants provides patients with a fixed restoration immediately after surgery, which has a significant positive impact on the quality of life of the patient.

The AGA for implant placement within the QZP delineates three pathways: extra-sinus, intra-sinus, and the lateral maxillary wall. To prevent an unfavorable emergence on the palate and substantial anterior extension, the implant placed anteriorly is commonly positioned along the ridge of the crest. Consequently, anterior ZIs often follow a pathway within a groove channel in the directional wall of the maxilla or outside the sinus. Due to the anatomical features of individuals with atrophic maxillary bone, the posterior implant is frequently placed outside the sinus or within the maxillary wall. Intra-sinus trajectories are often avoided due to the risk of sinusitis [24].

In a comprehensive meta-analysis and review, the occurrence of sinus issues in patients with intra-sinusal placement of ZIs was found to be 7.2%, significantly higher compared to patients with ZIs positioned extrasinusally, where the incidence was 1.8% [25].

The QZP demands a comprehensive understanding of anatomy and substantial experience because of the limited bone availability at the level of the maxillary and zygoma bone. Anatomical studies on the zygoma bone have revealed an average width of 4-8 mm and a length ranging from 25 to 32 mm. These measurements typically do not impose restrictions on placing two ZIs in most patients. Our study aligned with this, applying a width limitation of 15 mm consistent with these anatomical findings. However, in two cases where patients had thin zygomatic bones (measuring no less than 15 mm), there was a drilling incident resulting in slight penetration into the ocular cavity. Thankfully, this was managed by redirecting the drills without causing any clinical repercussions [26].

Recent research has concentrated on incorporating various guidance approaches to enhance the accuracy and safety of ZI placement during surgery. Surgical navigation technologies have notably enhanced the precision of ZI positioning, allowing for the integration of the restorative plan directly into the surgical site. For instance, Wu et al. found a slight 2.64° variation in 221 ZI implantations in atrophic edentulous patients utilizing a commercial dynamic navigation system [27]. In some cases, a modified customized template featuring multiple sleeves has been employed for the static surgical guide, demonstrating satisfactory accuracy in placing ZIs [28]. However, further clinical investigations are warranted to ascertain the effectiveness of both dynamic and static systems specifically for the QZP.

A recent extensive meta-analysis and review revealed that utilizing static and dynamic systems featuring visualization in real-time and monitoring approaches notably enhanced the clinical accuracy of ZI placement. Sinusitis has been identified as a prominent issue associated with the QZP and ZIs in general, occurring in 12.5% of our patients after an average of 10.3 years of ZI function. However, it's important to note that since not all patients with acute symptoms or possible chronic sinusitis detected through 1° imaging of the paranasal sinuses were thoroughly investigated, the actual prevalence of sinusitis might have been undervalued. While a definitive correlation between sinusitis and ZI trajectories couldn't be established, sinus infections did not show to lead to the reduction of ZI osseointegration in the zygomatic bone.

While this study offers valuable insights into the QZP for restoring edentulous patients with resorbed upper jaws, several limitations must be acknowledged. The retrospective design may introduce bias and limit causality. The sample size may not fully represent the broader population, and patient self-reporting could lead to underreporting of complications like sinusitis. The study's duration, though substantial, does not account for late-onset complications. Additionally, advancements in surgical techniques over time may affect the applicability of the results to current practices. Future prospective studies with larger, diverse populations, and standardized reporting are needed to confirm these findings and address these limitations.

## Conclusions

The QZP consistently provides excellent long-term success for restoring severely atrophic maxillae using four ZIs. This method offers a fixed solution where traditional implants fail and supports immediate loading through cross-arch stabilization, improving stability and functionality, and reducing treatment time. Furthermore, incorporating a distal cantilever can increase occlusal units and masticatory efficiency, potentially eliminating the need for additional procedures like ridge augmentation and sinus lifts. This study, despite a limited sample size, offers valuable insights into the long-term success and complications of ZIs for different prosthetic options.
